# Supplementary material for: Transcription Factor Prospero Homeobox 1 (PROX1) as a Potential Angiogenic Regulator of Follicular Thyroid Cancer Dissemination
Source: Int J Mol Sci. 2019 Nov 10;20(22):5619. doi: 10.3390/ijms20225619 (PMC6888435; doi:10.3390/ijms20225619)
Supplement: Supplementary file 1 [file ijms-20-05619-s001.pdf]

Supplementary

Table S1. Primers used for RT-qPCR

PROX1 (Prospero Homeobox 1)	5′ – CCAGCTCCAATATGCTGAAGACCTA – 3′<br>5′ – CATCGTTGATGGCTTGACGTG – 3′
MMP-1 (Matrix Metallopeptidase 1)	5' –CTGTCCCTGAACAGCCCAGTACTTA– 3'<br>5' –CTGGCCACAACTGCCAAATG– 3'
FGF2 (Fibroblast Growth Factor 2)	5′ - GGCTTCTTCCTGCGCATCCA – 3′<br>5′ – GCTCTTAGCAGACATTGGAAGA – 3′
MMP-3 (Matrix Metallopeptidase 3)	GAAATGAGGTACGAGCTGGATACC– 3′<br>5′ –ATGGCTGCATCGATTTTCCT– 3′
NUDT6 (Nudix Hydrolase 6)	5′ –GGCGAGCTGGACAGATTC– 3′<br>5′ –GCAGCAGGGGCAATAAATCG– 3′
BAIAP2 (BAI1 Associated Protein 2)	5′ –AAGTCCACAGGCAGATCCAG– 3′<br>5′ –GCCTTTGCTCCTTTGCTCAG– 3′
VEGFC (Vascular Endothelial Growth Factor C)	5′ –GCCACGGCTTATGCAAGCAAAGAT– 3′<br>5′ –AGTTGAGGTTGGCCTGTTCTCTGT– 3′
ANGPT1 (Angiopoietin 1)	5′ –GAAGGGAACCGAGCCTATTC– 3′<br>5′ –AGCATCAAACCACCATCCTC– 3′
KDR (Kinase Insert Domain Receptor)	5′ –AGGAGAGCGTGTCTTTGTGG– 3′<br>5′ –GCCTGTCTTCAGTTCCCCTC– 3′
VEGFA (Vascular Endothelial Growth Factor A)	5′ –CTTGCCTTGCTGCTCTACCT– 3′<br>5′ –AAGATGTCCACCAGGGTCTC– 3′
PLAT (Plasminogen Activator, Tissue Type)	5′ –AGGAGAGCGTGTCTTTGTGG– 3′<br>5′ –GCCTGTCTTCAGTTCCCCTC– 3′
MDK (Midkine)	5′ –CCTGCAACTGGAAGAAGGAG– 3′<br>5′ -- CTTTCCCTTCCCTTTCTTGG– 3′
ADAMTS9 (ADAM Metallopeptidase With Thrombospondin Type 1 Motif 9)	5′ –ACGAAAAACCTGCCGTAATG– 3′<br>5′ –TCAGAGTCTCCATGCACCAG– 3′
TIMP3 (TIMP Metallopeptidase Inhibitor 3)	5′ –CTGACAGGTCGCGTCTATGA– 3′<br>5′ –AGTCACAAAGCAAGGCAGGT– 3′
ACTB (Beta Actin)	5′ – GCCGAGGACTTTGATTGC – 3′<br>5′– CTGTGTGGACTTGGGAGAG – 3′

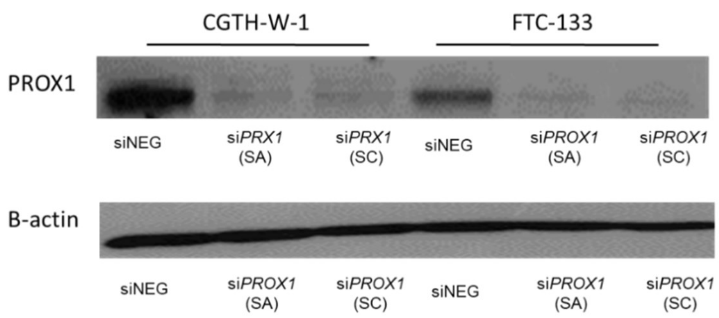

Figure S1. Efficient silencing of PROX1 in CGTH-W-1 and FTC-133 cells. Western blotting analysis shows a decrease in PROX1 protein level by targeting with siRNAs purchased from Santa Cruz (SC) and Sigma-Aldrich (SA) in both CGTH-W-1 and FTC-133 cell line. Beta-actin was used as a loading control of protein lysates.

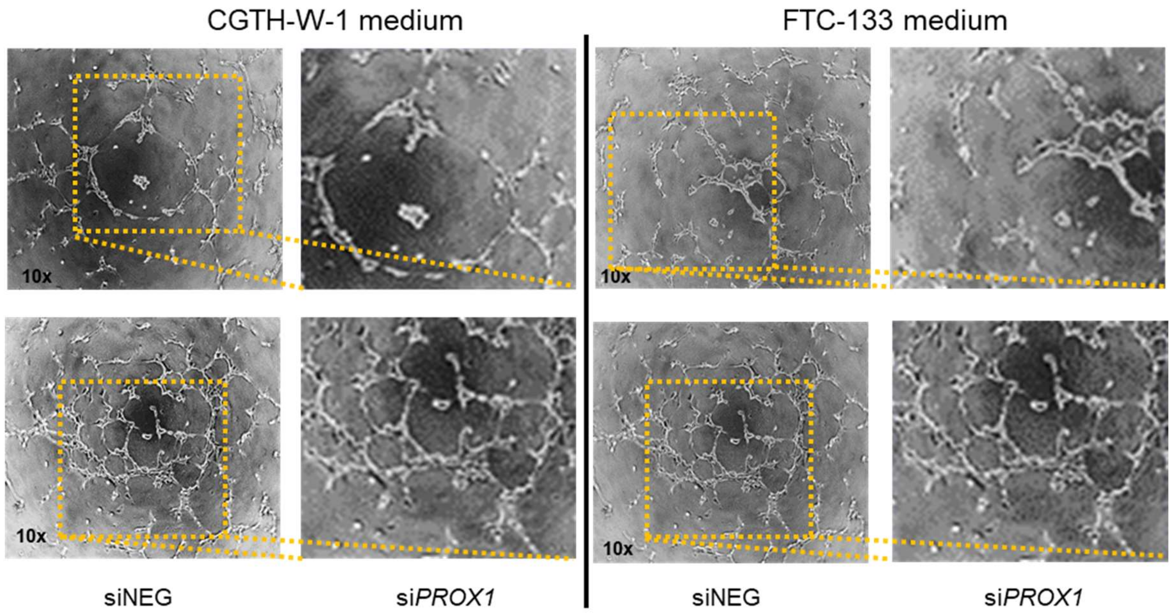

Figure S2. The tube formation assay. The silencing of PROX1 in CGTH-W-1 and FTC-133 cells enhances the angiogenesis *in vitro* of endothelial cells. HUVECs were cultured in 96-well plates coated with a semi-solid Matrigel. Cells were cultivated in conditioned medium collected from CGTH-W-1 and FTC-133 cells after silencing of PROX1 (with siRNA (SC)) and compared to HUVECs in medium collected from cells treated with siNEG. The vascularization effect was assessed under a light microscope after 5 h incubation; original magnifications: 10x.

Table S2. The list of angiogenic genes, which are significantly changed in the CGTH-W-1 cell line after the PROX1 knockdown using siPROX1from Sigma-Aldrich (SA) and Santa Cruz (SC). In the table, the upregulated genes are colored red, whereas the downregulated genes are colored green.

**gene symbol**	**gene name**	***p* value**	**fold change (SA)**	***p* value**	**fold change (SC)**
MMP1	matrix metallopeptidase 1 (interstitial collagenase)	0,0162	4,0250	0,0001	2,3912

FGF2	fibroblast growth factor 2 (basic)	0,0000	2,2940	0,8264	0,9647
MMP3	matrix metallopeptidase 3 (stromelysin 1, progelatinase)	0,0007	2,0390	0,0004	1,7780
IL24	interleukin 24	0,0065	1,9427	0,0031	1,7074
MCTS1	malignant T cell amplified sequence 1	0,0138	1,9280	0,0227	1,5566
LGALS1	lectin, galactoside-binding, soluble, 1	0,0000	1,7967	0,0105	1,4510
BRMS1	breast cancer metastasis suppressor 1	0,0006	1,7679	0,0174	1,4405
DKK1	dickkopf homolog 1 (Xenopus laevis)	0,0038	1,7620	0,0027	1,6146
KRT18	keratin 18	0,0047	1,6500	0,0050	1,5256
PTTG1	pituitary tumor-transforming 1	0,0002	1,6245	0,0077	1,5131
TK1	thymidine kinase 1, soluble	0,0009	1,6210	0,0119	1,3597
PCNA	proliferating cell nuclear antigen	0,0000	1,6041	0,0010	1,5668
NUDT6	nudix (nucleoside diphosphate linked moiety X)-type motif 6	0,0262	1,5930	0,7389	1,0610
EP300	E1A binding protein p300	0,0494	1,5706	0,0001	1,9584
MTOR	mechanistic target of rapamycin (serine/threonine kinase)	0,0012	1,5385	0,0043	1,4173
BAIAP2	BAI1-associated protein 2	0,0029	1,5350	0,0010	1,5799
HRAS	v-Ha-ras Harvey rat sarcoma viral oncogene homolog	0,0378	1,5336	0,0563	1,3573
MCAM	melanoma cell adhesion molecule	0,0031	1,5174	0,0001	1,8458
NRG1	neuregulin 1	0,0174	1,5150	0,0001	2,4956
KISS1	KiSS-1 metastasis-suppressor	0,0246	1,5076	0,0076	1,6321
NRAS	neuroblastoma RAS viral (v-ras) oncogene homolog	0,0023	1,4850	0,0048	1,4279
CDKN3	cyclin-dependent kinase inhibitor 3	0,0128	1,4600	0,0262	1,3771
VEGFC	vascular endothelial growth factor C	0,0083	1,4300	0,0572	1,2478
ANGPT1	angiopoietin 1	0,0463	1,3855	0,8629	0,9194
BDKRB1	bradykinin receptor B1	0,0429	1,3589	0,0130	0,7567
PTK2	PTK2 protein tyrosine kinase 2	0,0126	1,3504	0,0057	1,4274
PLAUR	plasminogen activator, urokinase receptor	0,0397	1,3303	0,0195	0,7450
ID1	inhibitor of DNA binding 1, dominant negative helix-loop-helix protein	0,0033	1,3029	0,0001	1,6678
ADAMTS9	ADAM metallopeptidase with thrombospondin type 1 motif, 9	0,2651	0,7955	0,4425	0,6736
CTNNA1	catenin (cadherin-associated protein), alpha 1, 102kDa	0,0480	0,7880	0,0346	0,7813

NRP2	neuropilin 2	0,0460	0,7516	0,0061	0,6989
HMGA1	high mobility group AT-hook 1	0,0373	0,7420	0,0052	0,6404
POFUT1	protein O-fucosyltransferase 1	0,0386	0,7291	0,1076	0,8194
PLXNA1	plexin A1	0,0217	0,7124	0,0013	0,6437
MTA1	metastasis associated 1	0,0152	0,7050	0,0871	0,8059
CXCL12	chemokine (C-X-C motif) ligand 12	0,0005	0,6937	0,0018	0,6906
MDK	midkine (neurite growth-promoting factor 2)	0,0032	0,6890	0,0635	0,7583
THBS1	thrombospondin 1	0,0051	0,6840	0,0559	0,5449
SFRP1	secreted frizzled-related protein 1	0,0012	0,6694	0,1859	0,7198
MVP	major vault protein	0,0052	0,6658	0,0010	0,6055
FN1	fibronectin 1	0,0002	0,6577	0,0365	1,3113
HTATIP2	HIV-1 Tat interactive protein 2, 30kDa	0,0557	0,6468	0,0589	0,6058
COL18A1	collagen, type XVIII, alpha 1	0,0127	0,6138	0,0104	0,6892
CLIC4	chloride intracellular channel 4	0,0000	0,6108	0,0000	0,5256
FOSL1	FOS-like antigen 1	0,0000	0,5829	0,0422	0,6727
PTEN	phosphatase and tensin homolog	0,0451	0,5769	0,0001	0,4454
POSTN	periostin, osteoblast specific factor	0,0299	0,5662	0,0259	0,6744
EGR1	early growth response 1	0,0075	0,5382	0,0008	0,5469
SDC2	syndecan 2	0,0003	0,5366	0,0000	0,5178
TIMP3	TIMP metallopeptidase inhibitor 3	0,0000	0,5332	0,0161	0,7486
AHR	aryl hydrocarbon receptor	0,0000	0,5176	0,0531	0,7561
IGFBP7	insulin-like growth factor binding protein 7	0,0029	0,4848	0,0028	0,5675
FTH1	ferritin, heavy polypeptide 1	0,0000	0,4347	0,0024	0,7104
LPAR1	lysophosphatidic acid receptor 1	0,0000	0,4239	0,0061	0,6825
PLAT	plasminogen activator, tissue	0,0000	0,3460	0,0001	0,3460

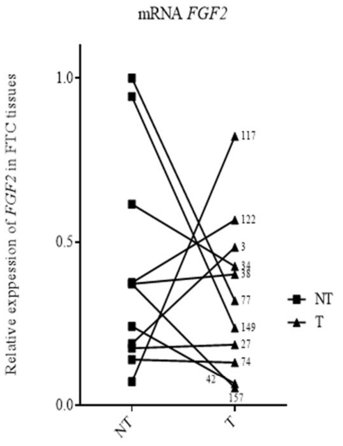

Relative expression of *FGF2* in FTC tissues	Non-tumoral	Tumoral
77	1,000	0,321
74	0,142	0,132
34	0,616	0,426
38	0,373	0,401
27	0,176	0,187
42	0,243	0,067
117	0,072	0,822
3	0,190	0,484
122	0,376	0,567
149	0,944	0,238
157	0,372	0,054

a

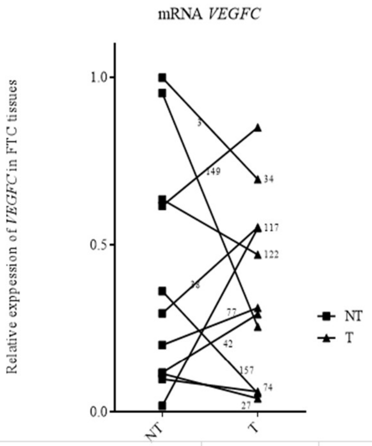

Relative expression of VEGFC in FTC tissues	Non-tumoral	Tumoral
77	0,202	0,313
74	0,099	0,061
34	1,000	0,697
38	0,296	0,554
27	0,115	0,041
42	0,119	0,294
117	0,019	0,551
3	0,954	0,257
122	0,637	0,472
149	0,617	0,852
157	0,363	0,057

c

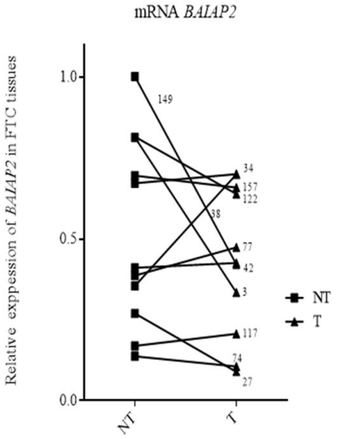

d

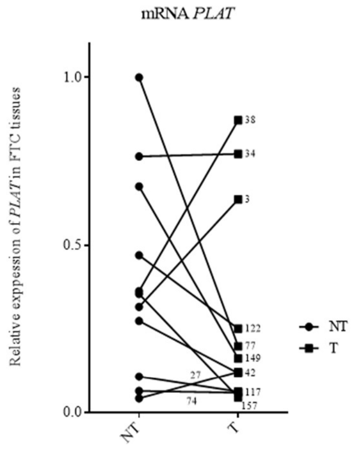

Relative expression of *BAIAP* in FTC tissues	Non-tumoral	Tumoral
77	0,387	0,474
74	0,138	0,107
34	0,672	0,700
38	0,355	0,699
27	0,271	0,091
42	0,411	0,426
117	0,170	0,208
3	0,813	0,335
122	0,815	0,639
149	1,001	0,422
157	0,695	0,658

Relative expression of *PLAT* in FTC tissues	Non-tumoral	Tumoral
77	1,000	0,198
74	0,107	0,062
34	0,764	0,772
38	0,363	0,872
27	0,042	0,121
42	0,274	0,118
117	0,064	0,059
3	0,316	0,636
122	0,470	0,251
149	0,675	0,162
157	0,354	0,045

Figure S3. Angiogenic factors: (a) *FGF2*, (b) *VEGFC*, (c) *BAIAP2*, (d) *PLAT* are differentially expressed in FTCs and normal thyroid tissues. The relative expression level of mRNAs in FTC tissues and non-tumoral tissues were analyzed by RT-qPCR and normalized against the housekeeping gene: *ACTB.*

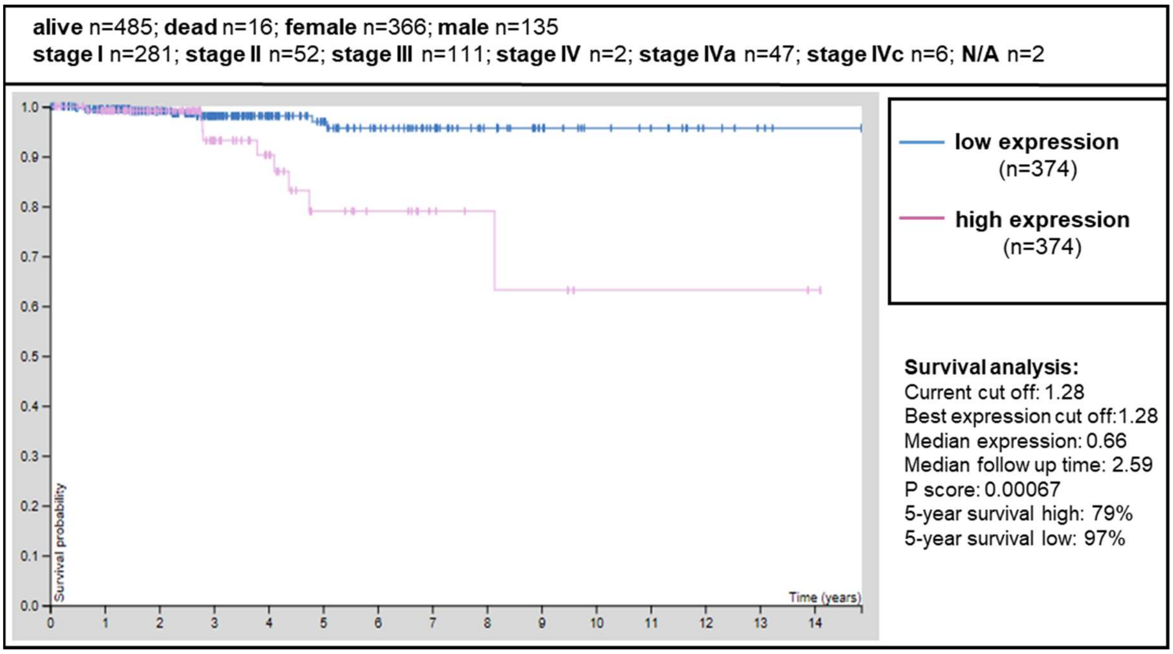

Figure S4. Correlation of PROX1 expression with the survival of patients with PTC. Data analysis obtained using The Human Protein Atlas https://www.proteinatlas.org/ENSG00000117707-PROX1/pathology/thyroid+cancer.

*P score: log-rank *p* value for Kaplan-Meier plot showing results from analysis of correlation between mRNA expression level and patient survival

*5-year survival for patients with higher / lower expression than the expression cutoff.
